# Real‐time *ex vivo* perfusion of human lymph nodes invaded by cancer (REPLICANT): a feasibility study

**DOI:** 10.1002/path.5367

**Published:** 2019-12-22

**Authors:** Rachel Barrow‐McGee, Julia Procter, Julie Owen, Natalie Woodman, Cristina Lombardelli, Ashutosh Kothari, Tibor Kovacs, Michael Douek, Simi George, Peter A Barry, Kelvin Ramsey, Amy Gibson, Richard Buus, Erle Holgersen, Rachael Natrajan, Syed Haider, Michael J Shattock, Cheryl Gillett, Andrew NJ Tutt, Sarah E Pinder, Kalnisha Naidoo

**Affiliations:** ^1^ Toby Robins Breast Cancer Now Research Centre, Breast Cancer Research Division The Institute of Cancer Research London UK; ^2^ King's Health Partners Cancer Biobank, Guy's Comprehensive Cancer Centre London UK; ^3^ Guy's and St. Thomas' Hospital NHS Foundation Trust London UK; ^4^ School of Cancer and Pharmaceutical Sciences King's College London, Guy's Comprehensive Cancer Centre London UK; ^5^ Department of Cellular Pathology, Guy's and St. Thomas' Hospital NHS Foundation Trust London UK; ^6^ Royal Marsden Hospital London UK; ^7^ Ralph Lauren Centre for Breast Cancer Research Royal Marsden Hospital London UK; ^8^ British Heart Foundation Centre of Research Excellence King's College London, St Thomas' Hospital London UK

**Keywords:** lymph node metastasis, breast cancer, immuno‐oncology, normothermic perfusion

## Abstract

Understanding how breast cancer (BC) grows in axillary lymph nodes (ALNs), and refining how therapies might halt that process, is clinically important. However, modelling the complex ALN microenvironment is difficult, and no human models exist at present. We harvested ALNs from ten BC patients, and perfused them at 37 °C *ex vivo* for up to 24 h. Controlled autologous testing showed that ALNs remain viable after 24 h of *ex vivo* perfusion: haematoxylin and eosin‐stained histological appearance and proliferation (by Ki67 immunohistochemistry) did not change significantly over time for any perfused ALN compared with a control from time‐point zero. Furthermore, targeted gene expression analysis (NanoString PanCancer IO360 panel) showed that only 21/750 genes were differentially expressed between control and perfused ALNs (|log_2_ FC| > 1 and *q* < 0.1): none were involved in apoptosis and metabolism, but rather all 21 genes were involved in immune function and angiogenesis. During perfusion, tissue acid–base balance remained stable. Interestingly, the flow rate increased (*p* < 0.001) in cancer‐replaced (i.e. metastasis occupied more than 90% of the surface area on multiple levels) compared to cancer‐free nodes (i.e. nodes with no metastasis on multiple sections). *CXCL11* transcripts were significantly more abundant in cancer‐replaced nodes, while *CXCL12* transcripts were significantly more abundant in cancer‐free nodes. These cytokines were also detected in the circulating perfusate. Monoclonal antibodies (nivolumab and trastuzumab) were administered into a further three ALNs to confirm perfusion efficacy. These drugs saturated the nodes; nivolumab even induced cancer cell death. Normothermic ALN perfusion is not only feasible but sustains the tumour microenvironment *ex vivo* for scientific investigation. This model could facilitate the identification of actionable immuno‐oncology targets. © 2019 The Authors. *The Journal of Pathology* published by John Wiley & Sons Ltd on behalf of Pathological Society of Great Britain and Ireland.

## Introduction

The ethical constraints around harvesting human tissue make model development difficult 1. In breast cancer (BC), the number of involved axillary lymph nodes (ALNs), and the size of each nodal metastasis, is intimately related to patient prognosis; as the volume of cancer in the nodes increases, survival proportionately decreases 2, 3, 4, 5, 6. Furthermore, primary and metastatic tumours may respond differently to therapeutic agents 7, 8, 9, 10.

At least 20% of oestrogen receptor (ER)‐positive and HER2‐positive BCs reportedly become triple‐negative at metastatic sites, including ALNs 9. The relevance of this to emerging immunotherapies is unclear, particularly since these drugs may be most relevant to the treatment of lymphocyte‐enriched triple‐negative BC 11. How the endogenous lymphoid population within metastatic ALNs interacts with immune‐checkpoint inhibitors (ICIs) has yet to be determined. In fact, most immuno‐oncology markers have only been evaluated in primary, not metastatic, sites. Studies show that programmed death‐ligand 1 (PD‐L1) expression (by tumour cells, and not immune cells) is seen in 12–21% of primary BCs; is enriched in triple‐negative BC (19–33%); significantly correlates with ALN metastasis; and adversely affects survival 12, 13, 14, 15, 16. Furthermore, how these markers change following ICI administration in patient samples is unknown.

For these reasons, pathological examination of every surgically removed ALN is mandated so that the multidisciplinary team (MDT) can decide if systemic therapies are indicated 5. Thus, finding a cohort of patients in which even one entire ALN can be donated to research is challenging. As a result, no human models of nodal metastasis exist at present 17, and the role of the human ALN, with its unique ability to bring tumour and immune cells together, in either facilitating or hindering metastasis has not been robustly investigated.

Normothermic *ex vivo* perfusion is a well‐established technique routinely used to keep donor organs viable for transplantation into a suitable recipient 18, 19. In the laboratory, in addition to other methods 20, 21, 22, the modified Langendorff system has long been used to investigate heart disease in rodent models 23. We postulated that this technique could be adapted to keep human ALNs viable *ex vivo* for scientific investigation.

This feasibility study was subject to certain ethical constraints. First, ALNs were only harvested from BC patients who were having an ALN dissection (ALND) as standard of care. Thus, patients had a high burden of nodal disease. Second, subsequent formalin fixation was a diagnostic requirement, and surplus tissue was released for research purposes only once the final pathology report had been issued. Finally, nothing that could potentially damage the tissue could be administered through the perfusion circuit. Despite these limitations, we have shown that *ex vivo* normothermic perfusion of human ALNs is feasible for translational research.

## Materials and methods

### Patient samples

Tissue samples were obtained through the King's Health Partners (KHP) Cancer Biobank (Research Ethics Committee No: 18/EE/0025). The number of positive nodes identified pre‐operatively had to be such that removing one or two for perfusion would not affect subsequent treatment recommendations by the MDT. Patients with only one positive ALN, or those who had had an excellent/good response to neoadjuvant chemotherapy such that accurate clinical assessment of nodal burden was difficult or that histological assessment would be problematic, were excluded. Table 1 outlines the clinical characteristics of enrolled patients (*n* = 13).

**Table 1:** Clinico‐pathological characteristics of the 13 patients enrolled in the study

Parameter	Distribution
Age, years	
< 50	1 (8%)
≥ 50	12 (92%)
Grade 49	
1	0 (0%)
2	6 (46%)
3	7 (54%)
Tumour type	
No special type (NST)	12 (92%)
Lobular	1 (8%)
Lymphovascular invasion	
Definite	6 (46%)
Negative/probable	7 (54%)
Lymph node status	
pN0/ypN0	1 (8%)
pN1/ypN1	4 (30%)
pN2/ypN2	6 (46%)
pN3	2 (16%)
Neoadjuvant therapy	
Endocrine	1 (8%)
Chemotherapy	4 (30%)
None	8 (62%)
ER status	
Negative (Allred score < 3)	3 (23%)
Positive	10 (77%)
PR status	
Negative (Allred score < 3)	5 (38%)
Positive	6 (46%)
Unknown	2 (16%)
HER2 status	
Negative	10 (77%)
Positive (3+ by IHC or 2+ and FISH‐amplified)	3 (23%)
Triple‐negative status	
Non‐triple‐negative	10 (77%)
Triple‐negative	3 (23%)
Completion clearance	
Yes	7 (54%)
No	6 (46%)

### ALN harvest

Fresh ALND specimens were collected and anonymised by the KHP Cancer Biobank prior to harvest. ALNs were palpated to assess whether overt metastasis was present or not and dissected out intact with blunt‐end forceps at room temperature (i.e. the entire node with 1–2 mm of attached fat was removed). The feeding artery was then cannulated with either a 24‐ or a 26‐gauge intravenous catheter (SURFLO®; VWR, Lutterworth, UK), which was secured with a silk suture (Resolon™; Resorba, Nuremberg, Germany; supplementary material, Figure S1). The node was then attached to the perfusion circuit.

### *Ex vivo* perfusion

Each ALN was perfused at a constant pressure 23, 24 and temperature (37 °C) with filtered Krebs–Henseleit solution 25, using a peristaltic pump (Gilson, Dunstable, UK), gassed with 95% oxygen–5% carbon dioxide to a pH of 7.4. Constant pressure perfusion was chosen over constant flow as this is more physiological (i.e. tissues have this *in vivo*) 23. Perfusion pressure was determined by the intrinsic resistance of each ALN, and was set/held as soon as buffer seeped out of the cut vessels radiating out of the node. The first two 4‐h samples underwent single‐pass perfusion. The subsequent eight samples underwent 1 h of single‐pass perfusion, after which fluid was recirculated and replenished four‐hourly. The circulating fluid percolating through the ALNs (‘perfusate’) was pumped out of the Petri dish continuously. Single‐pass perfusion was used when drugs were administered to the node. Between samples, all tubing (Altec Extrusions Ltd, St Austell, UK), three‐way taps (Bunzl Healthcare, London, UK), cannulae, and Petri dishes were discarded; glassware was autoclaved; and the system wiped down with 70% ethanol.

### Real‐time measurements during *ex vivo* perfusion

Pressure and flow readings were recorded via a blood pressure (BP) transducer using LabChart software Version 8.1.6 (AD Instruments, Oxford, UK) 23, 24. Before each experiment, the pump controller was calibrated using the simple two‐point calibration built into the LabChart data‐logger. Pressure readings were calibrated by attaching a sphygmomanometer (Omron, Milton Keynes, UK) to the BP transducer to establish a recording range between 0 and 220 mmHg. Thereafter, flow was calibrated by measuring the amount of fluid collected over 1 min at the lowest and highest pump speeds. Pressure was measured to a resolution of ±1 mmHg and flow rate to a resolution of ± 0.1 ml/min. ‘Blood gas’ readings were measured from the perfusate using CG4+ cartridges read with an i‐STAT handheld analyser (Abbott Point of Care Inc, Maidenhead, UK) 26. Glucose readings were taken with a glucometer (WaveSense JAZZ; AgaMatrix Europe Ltd, Didcot, UK).

### Targeted therapeutic agents

Nivolumab (1 μg; Bristol‐Myers Squibb, Uxbridge, UK) and trastuzumab (0.8 μg; Stratech Scientific, Ely, UK) were administered through the circuit in Krebs–Henseleit buffer for 60 and 90 min, respectively. Perfusion with Krebs–Henseleit buffer alone was then resumed. For nivolumab, normal saline was used as a vehicle control; for trastuzumab, phosphate‐buffered saline.

### Primary end‐points

Following perfusion, ALNs were sliced fresh and then placed into 10% neutral buffered formalin (NBF) within 10 min of removal from the perfusion circuit (by KN) to ensure good fixation. Samples remained in NBF for 8–12 h, during which time they were repatriated to the rest of the diagnostic specimen for handling and reporting. Once reported, surplus tissue (with relevant clinico‐pathological information) was anonymised by the KHP Cancer Biobank before being released to researchers for retrospective analysis. Perfused ALNs were compared with an autologous (i.e. taken from the same patient) matched control (‘baseline control’) that was of the same disease status (i.e. either both cancer‐replaced or both cancer‐free). The baseline control ALNs were formalin‐fixed at time‐point zero, i.e. as soon as they had been surgically removed from the patient. ALNs perfused with a therapeutic antibody were additionally compared with an autologous ALN that had received an appropriate drug vehicle in tandem (‘vehicle control ALN’).

Viability was defined as maintenance of normal ALN architecture with more than 90% cellular viability at each time‐point on blinded whole‐section microscopic examination. Cellular proliferation was assessed using Ki67 immunohistochemistry 27. The presence of metastatic tumour deposits was confirmed microscopically. If tumour occupied more than 90% of the total surface area (TSA) of a node on multiple levels, it was termed ‘cancer‐replaced’; if tumour occupied less than 90% of the TSA of a node on multiple levels (i.e. micro‐ or macro‐metastases 28 not completely replacing the node), it was classified as having ‘discrete tumour deposits’; if no tumour cells were identified on multiple levels, the node was deemed ‘cancer‐free’. If present, tumour necrosis was assessed as an absolute percentage of the TSA on whole‐section microscopic analysis.

### Sample size determination

Based on the above‐mentioned definition of viability, with an anticipated absolute difference of 10 in means and a standard deviation of 5, in order to achieve 90% (beta = 0.1) power for alpha = 0.05 (to reject null hypothesis), we estimated (using https://clincalc.com/stats/samplesize.aspx) that we required a minimum of five samples per group assuming equal‐sized groups. Ultimately, ten nodes per group were analysed.

### Immunohistochemistry

Sections cut at 4 μm thickness were immunostained using commercially available antibodies for Ki67 [1:400, clone MIB‐1 (M7240); Dako, Agilent, Stockport, UK], pan‐cytokeratin [1:200, clone MNF‐116 (M0821); Dako], PD‐L1 (pre‐diluted; Dako PD‐L1 IHC 22C3 PharmDx performed on Autostainer Link 48), and HER2 [pre‐diluted; PATHWAY® anti‐HER2/neu (45B) Rabbit Monoclonal Primary Antibody; Ventana Systems UK, Salisbury, UK] in a UKAS accredited laboratory. Ki67 and pan‐cytokeratin immunostaining was performed on a Leica Bond‐III automated stainer (Leica, Milton Keynes, UK). Images were taken using an Optika Pro 3 digital camera and an Olympus 3X51 microscope and processed in Adobe Photoshop CC (version 2017.1.1) to adjust the white balance.

### Ki67 analysis

Two blinded analyses were performed. First, a pathologist (KN) scored 400 cells across four high‐power fields in inter‐follicular regions (i.e. germinal centres were avoided). Ki67 was also analysed using an alternative method in which germinal centres, which form Ki67 hotspots in cancer‐free lymph nodes, were assessed (by RBM). An average of more than 3000 cells were included over ten high‐power fields per case using FIJI/ImageJ software 29, 30. The values obtained with both methods were converted to an absolute percentage for comparative statistics.

### RNA extraction

Two serial 10‐μm‐thick sections of each ALN were deparaffinised with xylene and rehydrated in 100% and then 70% ethanol in water. RNA was extracted using an RNeasy FFPE Kit (Qiagen, Manchester, UK) according to the manufacturer's instructions. RNA quantity and quality were evaluated by Nanodrop 2000 (Thermo Fisher Scientific, London, UK) and an Agilent 4200 TapeStation. All 13 patient samples passed quality control, highlighting the importance of the short formalin fixation and storage times of the study. No samples were excluded from the final analysis.

### NanoString nCounter assay

The PanCancer IO360 Gene Expression Panel 31 (750 targeted and 20 reference genes) was used to assess whether perfusion affected targeted (immuno‐oncology) gene expression. This technology works robustly on small quantities of formalin‐fixed, paraffin‐wax embedded (FFPE) tissue, which complied with study ethics. For each sample, 100–400 ng of RNA was analysed; this was calculated as 80 ng of RNA adjusted for the % of RNA fragments consisting of 50–300 nucleotides of the total RNA. Samples were run on the NanoString® nCounter® SPRINT Profiler (NanoString® Technologies, Seattle, WA, USA) according to the manufacturer's protocols.

### Cytokine ELISA

CXCL11 and CXCL12 were quantified in triplicate in the perfusate (end‐point) using Quantikine ELISA kits following the manufacturer's instructions (R&D Systems, Abingdon, UK). The optical density was determined using an Infinite® Pro 200 microplate reader (Tecan, Reading, UK) at 450 nm and readings obtained at 570 nm were subtracted to correct for optical imperfections in the plate.

### Data analysis

The NanoString® PanCancer IO 360 Gene Expression Panel was pre‐processed using R package NanoStringNorm 32 version 1.2.1. Data were assessed for batch effects using R package FactoMineR 33 version 1.39. Raw counts of endogenous genes were normalised using R package edgeR 34 version 3.20.9 with the TMM method.

Differential abundance of mRNA data was performed using R package *limma* (voom) 35 version 3.34.9. Perfused versus control lymph nodes were assessed using model: *∼condition + sample. pair*, and metastatic versus lymph nodes were assessed using model: *∼0 + condition*. Differentially abundant genes were defined as those with a Benjamini–Hochberg‐adjusted *P* value of less than 0.1 with an absolute log_2_ fold‐change (|log_2_ FC|) > 1. All visualisations were generated using custom graphics libraries in R statistical environment version 3.4.0.

### Statistical analysis

Two‐way ANOVA was used to assess the effect of tumour status on ALN flow rate over time. A two‐tailed Mann–Whitney test was used to assess differences in Ki67.

## Results

### Establishing a viable perfusion model

**Figure 1:**
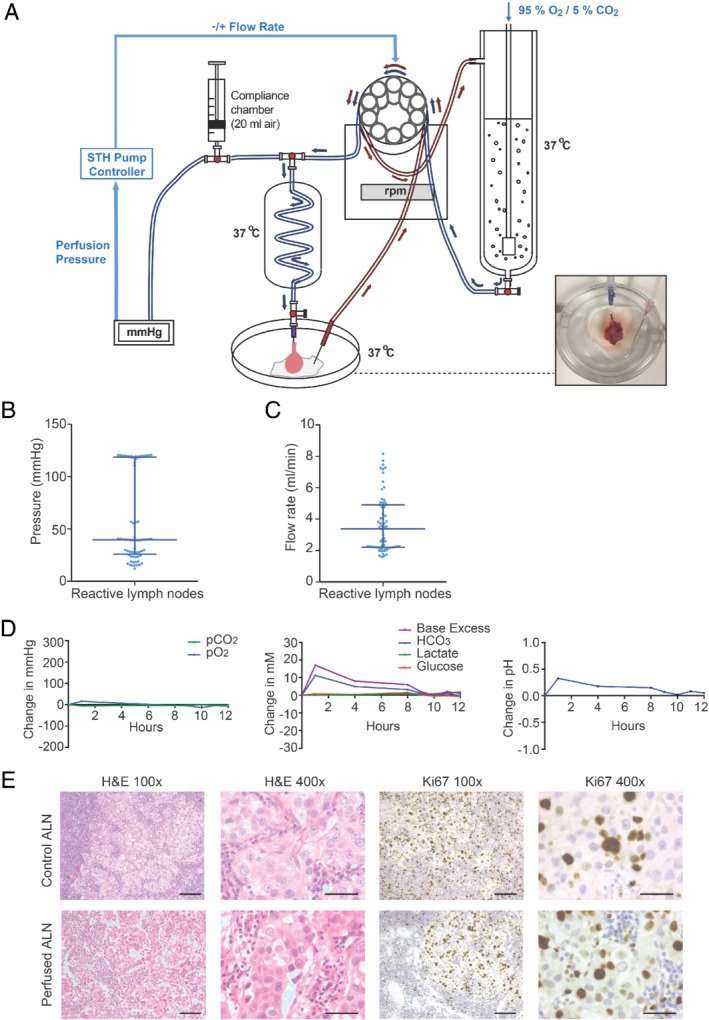
Setting up a viable perfusion model. (A) Diagram of the perfusion circuit. Cannulated axillary lymph nodes (ALNs; photograph, bottom right) were perfused at 37 °C as shown. (B, C) Defining the flow rate through cancer‐free human ALNs. Scatter plots show the median pressure (B) and flow rate (C) with interquartile range in cancer‐free perfused ALNs (*n* = 5 ALNs and patients; individual data points are the hourly pressure and flow rate readings for each of the five ALNs). (D, E) Example of the various data collected during each experiment, taken from a cancer‐replaced ALN after 12 h of perfusion (grade 3 no special type; oestrogen receptor‐positive; Her‐2‐negative; no lymphovascular invasion; pN2). (D) Acid–base status and metabolism were monitored regularly. Graphs show change over time. (E) Viability and proliferation (Ki67 immunohistochemistry) assessed on whole‐section examination following perfusion (scale bars: 50 μm). p = pathological nodal stage.

Perfusion was carried out for 4, 8, 12 or 24 h (Figure 1A). The median perfusion pressure through cancer‐free ALNs was 39.72 mmHg (Figure 1B; *n* = 5), and the median flow rate was 3.39 ml/min (Figure 1C). This did not appear to be affected by ALN size (supplementary material, Figure S2A) or perfusion pressure (supplementary material, Figure S2B).

Acid–base balance was monitored regularly 26. This remained stable over 24 h (Figure 1D and supplementary material, Figures S2–S7). Flow rates of ≤ 1 ml/min caused the perfusate to stagnate in the Petri dish, losing carbon dioxide to the atmosphere 36. Lactate levels did not rise concurrently however, suggesting that metabolism was unaltered.

**Figure 2:**
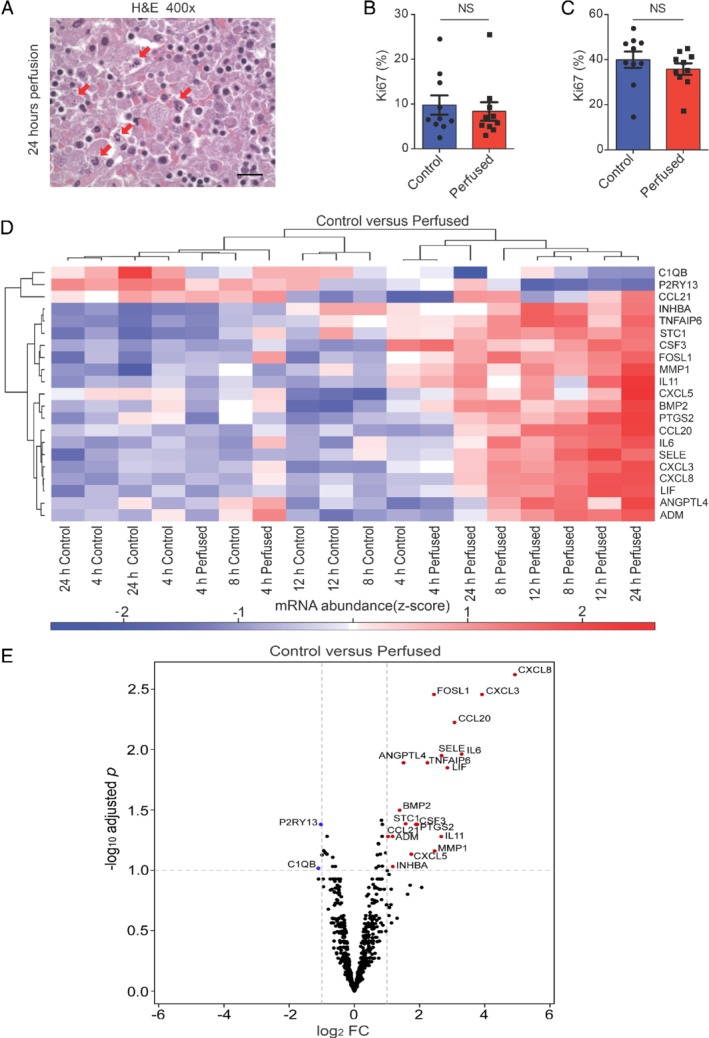
Samples remained viable for up to 24 h of perfusion. (A) Apoptosis became histologically evident at 24 h of perfusion. Arrows show apoptotic debris (scale bar: 20 μm). (B, C) Two Ki67 quantification methods showed that perfusion did not alter proliferation in axillary lymph nodes (ALNs; *n* = 10 patients) compared with baseline control ALNs (fixed at time‐point 0; mean Ki67 % with SEM). (D, E) Heatmap (D) and volcano plot (E) of the 21 significantly differentially expressed genes between perfused ALNs and baseline control ALNs (|log_2_ FC| > 1 and *q* < 0.1; blue, down‐regulated; red, up‐regulated). All the named genes are considered to be significantly differentially expressed: those with a blue circle were down‐regulated in perfused ALNs; those with a red circle were up‐regulated in perfused ALNs.

After each experiment, viability was confirmed in comparison to a matched autologous ‘baseline control’ ALN (fixed at time‐point zero) in at least two perfused ALNs before the perfusion time for subsequent samples was increased. This baseline control ALN was taken from the same patient and was of the same disease status (i.e. either both cancer‐replaced or both cancer‐free). All perfused ALNs (*n* = 10 nodes and patients) were viable on whole‐section histological haematoxylin and eosin examination (Figure 1E and supplementary material, Figures S2, S4–S7); there was no histological evidence of ischaemia or necrosis. However, scattered apoptotic debris (5% total ALN surface area) became visible after 24 h of perfusion (Figure 2A). Since our definition of viability allowed for 10% apoptosis/necrosis, this was considered negligible. Ki67 immunohistochemistry 27 showed equivalent proliferation in perfused and baseline control ALNs (representative picture, Figure 1E and Figure 2B,C).

No significant changes in gene expression were seen between the control and perfused samples (|log_2_ FC| > 1 and *q* < 0.05). When less stringent criteria were applied, only 21/750 (2.8%) significantly differentially expressed genes (DEGs) were seen in perfused ALNs compared with baseline control ALNs (|log_2_ FC| > 1 and *q* < 0.1; Figure 2D,E). All of these were involved in immune function and angiogenesis; none were related to apoptosis, proliferation, hypoxia, or metabolism.

These data demonstrate that human ALNs can be sustained for 24 h *ex vivo* using this model, with negligible effects on histology, proliferation, and targeted gene expression.

### Using real‐time measurements to understand ALN biology

Having established that perfusion did not alter ALN histology, we wanted to determine how/if the real‐time measurements taken during perfusion related to the disease status of each node. Some interesting differences were seen between cancer‐free and cancer‐replaced ALNs.

**Figure 3:**
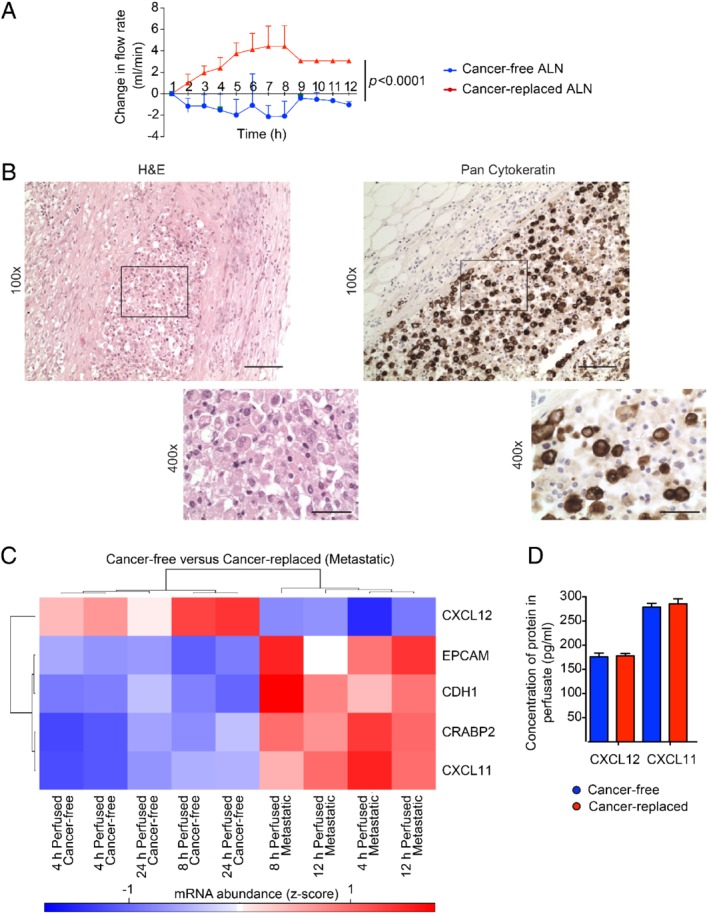
Using real‐time measurements to understand axillary lymph node (ALN) biology. (A) Cancer‐replaced ALNs (*n* = 3) showed a higher flow rate during perfusion than cancer‐free ALNs (*n* = 5) over time (two‐way ANOVA; *p* < 0.0001; mean with SD). (B) Cancer‐replaced ALN with pan‐cytokeratin‐positive cancer cells filling the subcapsular sinus (scale bars: 50 μm). (C) Heatmap of the five significantly differentially expressed genes between cancer‐free and cancer‐replaced (metastatic) perfused ALNs (|log_2_ FC| > 1 and *q* < 0.1; blue, down‐regulated; red, up‐regulated). (D) Concentration of CXCL12 and CXCL11 in the perfusate of cancer‐free and cancer‐replaced ALNs measured using an ELISA assay (*n* = 10, performed in triplicate).

In order to correct for any differences in perfusion pressures between samples, the change in flow rate from baseline was calculated over time (Figure 3A). This increased in cancer‐replaced ALNs but decreased in cancer‐free ALNs (*p* < 0.001). ALNs that had discrete tumour deposits behaved similarly to cancer‐free nodes (*n* = 2; data not shown). Histologically, cancer cells were seen in the subcapsular sinuses, as well as the parenchyma, of these cancer‐replaced nodes; these sinuses lead to efferent lymphatics (Figure 3B). The increase in flow rate in cancer‐replaced ALNs suggests that tissue resistance had decreased during the course of the experiment. We postulated that this could be due to a change in cytokine‐induced vasodilatation in the cancer‐replaced nodes, since NanoString analysis showed only five significantly differentially‐expressed genes (DEGs) between cancer‐free and cancer‐replaced nodes following FDR correction (|log_2_ FC| > 1 and *q* < 0.1, Figure 3C). Two (*EPCAM* and *CDH1*) reflect the presence of malignant epithelium in cancer‐replaced nodes and its absence in cancer‐free nodes. Of the remaining three genes, *CXCL11* and *CRABP2* were significantly up‐regulated in metastatic nodes, whilst *CXCL12* was significantly down‐regulated.

These results prompted us to assess if CXCL12 and CXCL11 protein could be detected in the perfusate by ELISA. Both were present (Figure 3D), confirming that these cytokines continue to be secreted during perfusion. However, no quantitative differences were seen. These proteins have very short half‐lives however, and this result could reflect the fact that the perfusate was kept at 37 °C for 4 h as the fluid recirculated, before being stored at −20 °C prior to analysis.

These data suggest that ALN colonisation 37 functionally alters the node during perfusion: the flow rate increased in cancer‐replaced, but not cancer‐free, nodes; and the cytokine expression profiles differed significantly at the transcript level. This putative association needs to be investigated in future experiments. However, these data show that the model can be used to investigate functional differences between cancer‐free and cancer‐replaced ALNs.

### Using therapeutic agents to evaluate perfusion efficacy and treatment effect

We wanted to confirm that fluid had permeated through the entire ALN during these experiments but were restricted ethically in what we could administer to the nodes. For this reason, therapeutic monoclonal antibodies were selected and administered through the circuit into ALNs from a further three patients. For each of these experiments, two ALNs were harvested from each patient: one was perfused with a targeted antibody and the other with an appropriate vehicle control. Both ALNs were perfused for 12 h to eliminate any perfusion‐related apoptosis (previously seen at 24 h). Finally, once fixed, these two perfused ALNs were again compared with an autologous baseline control ALN.

**Figure 4:**
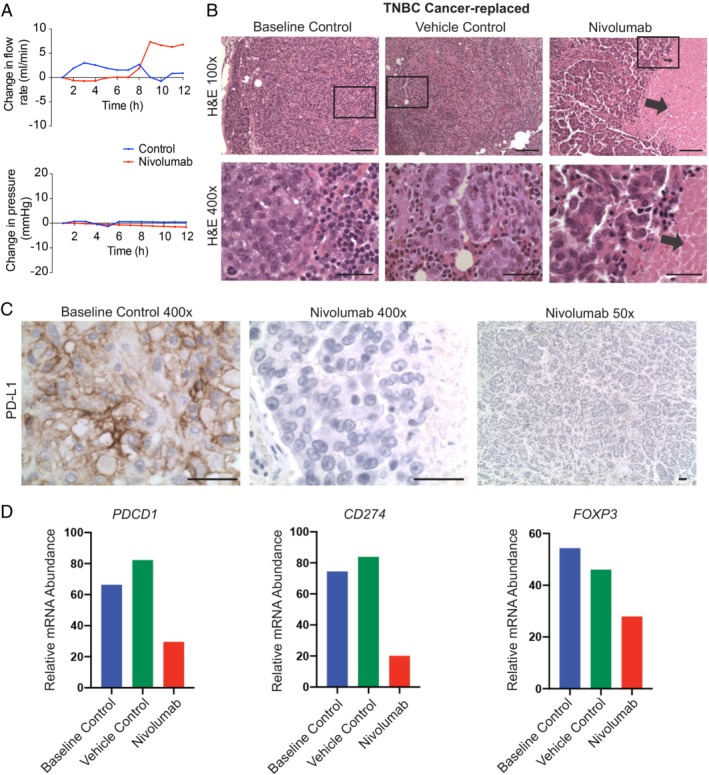
Nivolumab induced cancer cell death in a cancer‐replaced axillary lymph node (ALN). (A) Nivolumab infusion (1 μg over 60 min) resulted in an increased flow rate through an ALN from a patient with triple‐negative breast cancer (not seen in the vehicle control). (B) Histological cancer cell necrosis was observed in the nivolumab‐treated ALN (arrows), but not in the vehicle (normal saline) or baseline control ALN (fixed at time‐point 0; scale bars: 50 μm). (C) All tumour cells in the baseline control ALN were positive for PD‐L1. The nivolumab‐treated ALN was completely negative (scale bars: 50 μm). (D) NanoString IO360 analysis showed that *PDCD1* (PD‐1), *CD274* (PD‐L1), and *FOXP3* transcript were down‐regulated in the nivolumab‐treated node compared with both control ALNs.

First, we harvested two metastatic ALNs from a patient with triple‐negative BC who had received neoadjuvant carboplatin/paclitaxel chemotherapy. Since there is clinical evidence that ‘induction’ chemotherapy sensitises triple‐negative BC to the PD‐1 inhibitor nivolumab 38, 39, one node was perfused with nivolumab (1 μg 40) and the other with a vehicle control (normal saline) over 60 min. The flow rate in the nivolumab‐treated node rose after 8 h of perfusion (Figure 4A). This real‐time readout suggested a possible underlying biological change, which was confirmed histologically as scattered tumour necrosis (15% of the total area of the metastatic tumour; Figure 4B). Both the vehicle and the baseline control ALNs contained tumour but showed no evidence of necrosis (Figure 4B), indicating that necrosis was not an inherent feature of the metastatic disease. The tumour in the baseline control was strongly positive for PD‐L1 (Figure 4C; more than 50% of metastatic carcinoma cells showed strong, membranous expression). The nivolumab‐treated ALN, in contrast, was completely negative for PD‐L1 (Figure 4C; 0% of tumour cells showed strong, membranous expression). Whole‐section NanoString analysis showed that transcripts for *programmed cell death protein 1* (*PD‐1*; *PDCD1*), *programmed cell death 1 ligand 1 (PD‐L1*; *CD274*), and *Forkhead box P3* (*FOXP3*) were down‐regulated in the nivolumab‐treated node compared with the controls. Thus, it was interpreted that the drug had completely permeated the ALN. Two cancer‐free ALNs were harvested from a second patient with triple‐negative BC who, in contrast, had had a complete pathological response to neoadjuvant chemotherapy, i.e. had no metastatic disease present in the nodes. Again, one ALN was perfused with nivolumab (1 μg), and the other with a vehicle control (normal saline), over 60 min. Necrosis was not seen in this nivolumab‐treated ALN, suggesting that the necrosis observed in the previous patient sample was cancer cell‐specific.

**Figure 5:**
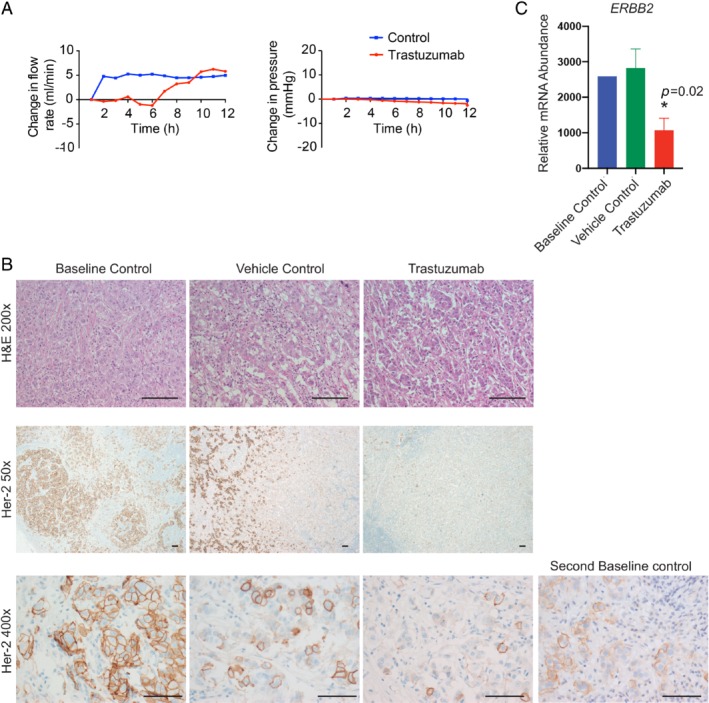
HER2 heterogeneity in a trastuzumab‐perfused axillary lymph node (ALN). (A, B) Trastuzumab infusion (0.8 μg over 60 min) through an ALN from a patient with HER2‐positive disease. (A) No drug‐specific changes in flow rate were seen. (B) HER2 expression was not seen in 85% of tumour cells in the trastuzumab‐treated ALN, confirming perfusion efficacy. The vehicle control (phosphate‐buffered saline) ALN also contained a small population of tumour cells that did not express HER2 (35%), as did one of the baseline control ALNs taken from the same patient. Thus, intra‐tumour heterogeneity was a feature of this tumour (scale bars: 50 μm). (C) NanoString IO360 analysis of four different whole sections from the baseline control and trastuzumab‐treated nodes showed the *ERBB2* transcript to be significantly down‐regulated (Mann–Whitney; *p* = 0.02; mean with SD).

Next, we harvested two metastatic ALNs from a treatment‐naïve patient with HER2‐positive disease. One was perfused with trastuzumab (0.8 μg 41), and the other with a vehicle control (PBS), over 90 min. No drug‐specific increase in flow rate (Figure 5A) or necrosis was seen. The metastatic baseline control ALN was strongly HER2‐positive (Figure 5B; 100% of tumour cells (3+ on immunohistochemistry 42). In contrast, 85% of cancer cells in the trastuzumab‐treated ALN were HER2‐negative (score = 1; Figure 5B). A similar decrease in staining has been seen in human clinical samples post‐neoadjuvant treatment (including trastuzumab) in both breast and gastric cancer 43, 44. Thus, these data suggest that the drug, once again, had completely permeated the ALN. However, 35% of cancer cells in the vehicle‐control ALN were also HER2‐negative (Figure 5B**)**. We therefore examined multiple sections of the primary carcinoma and all other positive nodes from the ALND to assess whether HER2 heterogeneity was present. One area of the primary breast tumour was HER2‐negative (Figure 5B) and two baseline control ALNs also contained HER2‐negative metastatic foci. Thus, the HER2‐negative foci in the vehicle control ALN may represent clonal divergence 9. NanoString analysis of multiple whole sections from the vehicle control and trastuzumab‐treated nodes (Figure 5C; *n* = 4 sections per node) confirmed that despite the presence of HER2 heterogeneity, *ERBB2* (*HER2*) transcript abundance was significantly higher in the untreated vehicle control ALN (*p* = 0.02). In other words, the amount of HER2‐negative tumour in the trastuzumab‐treated ALN was greater than in the control ALN (transcript and protein).

The monoclonal antibody data validated the perfusion technique, confirming that perfusate permeated entirely through the lymph nodes during perfusion. The decrease in PD‐1/PD‐L1 and HER2 expression following drug administration also suggests that these agents could bind to their cognate receptors in the model, seemingly inducing therapeutic effect.

## Discussion

We have shown, for the first time, that normothermic *ex vivo* lymph node perfusion is feasible for the purposes of scientific investigation. Nodal architecture (i.e. the tumour microenvironment) is maintained during perfusion and, importantly, the patient's own tissue (i.e. a matched autologous ALN) can be used as an analytical control. This makes the model particularly relevant for the preclinical evaluation of personalised therapies.

By comparing ten perfused with ten baseline control ALNs, we demonstrated that nodal tissue remains viable for up to 24 h of perfusion. The fact that apoptotic debris became visible at that time‐point probably reflects the use of crystalloid solution in the experiments; the circuit could be adapted for colloid or whole blood administration in the future to facilitate longer perfusion times.

ALN architecture and cellularity were maintained during perfusion. The complexity of the ALN microenvironment holds its own challenges in terms of analysis, particularly since formalin fixation was a diagnostic requirement. However, targeted immuno‐oncology analysis showed that few genes change during perfusion and that those that did were not involved in apoptosis, hypoxia, or metabolism.

The comparison of perfused ALN of different disease states (i.e. cancer‐free versus cancer‐replaced) was interesting. *CRABP2* has been shown to be up‐regulated in non‐perfused, fresh‐frozen metastatic ALNs from patients with ER‐positive disease (predominant in our cohort) 45. Furthermore, the expression of CRABP2 at the protein level has also been shown to correlate with a poor prognosis in BC 46. CXCL12 is known to facilitate BC metastasis to regional ALNs *in vivo*, via up‐regulation in normal human lymph nodes 47. Overall, the data from these various studies match our NanoString data and are a compelling indication that the molecular characteristics of the tissue are preserved during perfusion.

CXCL11 and CXCL12 protein were present in the sample perfusate. Interestingly, similar concentrations of CXCL11 have been found in the serum of patients with metastatic melanoma 48. In that study, however, high baseline levels of CXCL11 in the serum were associated with a significantly poorer outcome in patients treated with the CTLA‐4 inhibitor ipilimumab; the authors even propose that quantifying pretreatment levels of CXCL11 could help to identify which patients would benefit from immune‐checkpoint inhibitor therapy. This raises the possibility that the perfusate from the model that we describe might serve as a ‘serum proxy’ for immuno‐oncology biomarker identification in the future.

A limitation of the model in its current form is that one is uncertain of the definitive disease status of an ALN before perfusion. Whilst some large macro‐metastases (> 2 mm in size 28) may be palpable, the presence of smaller tumour deposits cannot be determined without histological examination. The real‐time data hold promise in circumventing this; the consistent rise in the flow rate through cancer‐replaced ALNs suggests that, if present, this could be used to infer large ALN tumour volume prior to fixation. Alternatively, the study design could be adapted to include a pre‐operative ALN ultrasound with or without a core needle biopsy that would establish the disease status of the node before excision, and if the ALN were clipped simultaneously, it could easily be identified for perfusion in the excision specimen.

The fact that therapeutic antibodies interacted with the cells within the node is promising. Both agents permeated the ALN, labelling tumour cells and validating the perfusion technique. The presence of HER2‐negative tumour cells in a vehicle control ALN highlights the importance of inter‐tumour heterogeneity (ITH) in invasive BC, which is a major consideration in modelling this disease. For this feasibility study, ITH did not preclude assessing viability. ITH probably will, however, affect how one asks both biological and therapeutic questions in this model system and will need to be accounted for in future experiments. The decrease in PD‐1/PD‐L1 seen in response to nivolumab administration is potentially exciting but should be interpreted with caution. The underlying mechanism is unclear, and the result needs to be replicated. This should not, however, detract from the primary aim of the experiment, i.e. confirming perfusion efficacy.

We have shown herein that normothermic *ex vivo* perfusion of human ALNs is feasible, and that the model can be used for hypothesis and intervention testing. We hope that the flexibility of this system will make it translationally relevant for the identification of actionable immuno‐oncology targets and biomarkers not only in BC but also in other cancers prone to lymph node metastasis, e.g. melanoma.

## Author Contributions Statement

KN conceived the study. RBM and KN designed the experiments, with advice from ANJT and SEP. RBM, PAB, KN, KR, ANJT, and SEP applied for ethical study approval. JO, NW, CL, SG, KN, SEP, and CG oversaw the collection, use, and storage of human tissue through the King's Health Partners Biobank. PAB and KR provided advice around surgical aspects of the study. AK, TK, and MD consented patients for the study and performed the surgeries. MJS provided guidance on setting up the perfusion circuit and advised on perfusion‐related issues arising from the experiments. KN dissected out ALNs and cannulated them for perfusion. RBM, JP, and KN perfused the ALNs used in the study and collected and analysed real‐time data during perfusion. KN and SEP analysed tissue samples histologically. KN and RBM quantified Ki67 immunohistochemistry. NW and CL cut curls, and RBM and JP extracted RNA, from tissue samples. AG and RB ran the NanoString analysis. SH, EH, RN, RBM, and KN analysed the NanoString data. RBM and KN wrote the manuscript, with critical input from all the other authors.

## Supporting information


**Figure S1.** Cannulation of an axillary lymph node (ALN)
**Figure S2.** The median flow rate through cancer‐free axillary lymph nodes (ALNs) was not affected by ALN size or perfusion pressure
**Figure S3.** Real‐time electrophysiological and biochemical readings taken from the perfusate
**Figure S4.** Axillary lymph nodes (ALNs) perfused for 4 h
**Figure S5.** Axillary lymph nodes (ALNs) perfused for 8 h
**Figure S6.** Axillary lymph nodes (ALNs) perfused for 12 and 4 h
**Figure S7.** Axillary lymph node (ALN) perfused for 24 h

## Data Availability

The anonymised NanoString dataset of gene expression changes is available on Zenodo.org (https://zenodo.org/record/3543693).
