# Supplementary material for: Real‐time ex vivo perfusion of human lymph nodes invaded by cancer (REPLICANT): a feasibility study
Source: J Pathol. 2019 Dec 22;250(3):262–74. doi: 10.1002/path.5367 (PMC7065097; doi:10.1002/path.5367)
Supplement: Supplementary file 1 — Figure S1. Cannulation of an axillary lymph node (ALN) Figure S2. The median flow rate through cancer‐free axillary lymph nodes (ALNs) was not affected by ALN size or perfusion pressure Figure S3. Real‐time electrophysiological and biochemical readings taken from the perfusate Figure S4. Axillary lymph nodes (ALNs) perfused for 4 h Figure S5. Axillary lymph nodes (ALNs) perfused for 8 h Figure S6. Axillary lymph nodes (ALNs) perfused for 12 and 4 h Figure S7. Axillary lymph node (ALN) perfused for 24 h [file PATH-250-262-s001.docx]

**Real-time *ex vivo* perfusion of human lymph nodes invaded by cancer (REPLICANT): a feasibility study**

Barrow-McGee *et al. J Pathol* DOI: 10.1002/path.5367

**Supplementary figures S1–S7**

**
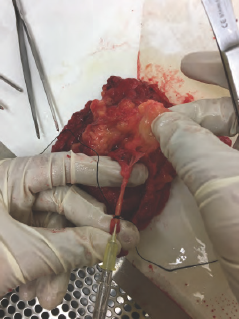
**

**Figure S1. Cannulation of an axillary lymph node (ALN).**

A fresh ALN (visible beneath the right thumb of the pathologist) being harvested from an axillary lymph node dissection specimen at room temperature. The feeding artery has been dissected free from the surrounding fat and a 24-gauge intravenous catheter has been inserted into the artery and secured with a suture. The node with cannulated vessel was then cut free from the rest of the specimen, placed in a Petri dish, and attached to the perfusion circuit.

**Figure S2. The median flow rate through cancer-free axillary lymph nodes (ALNs) was not affected by ALN size or perfusion pressure.**

(A, B) Two cancer-free ALNs were perfused for (A) 4 h (30 mm node) and (B) 24 h. Top-left panels: ALN histology. Representative microscopy images of haematoxylin and eosin- and Ki67-stained ALN sections at x 100 and x 400 magnification (scale bars: 50 µm). Top-right panels: graphs show the flow rate (top) and perfusion pressure (bottom) over time. Bottom panels: ‘blood gas’ readings were measured from the ALN perfusate. Graphs show the change in pCO_2_, pO_2_, base excess, HCO_3_, lactate, glucose, and pH readings, calculated from the first reading taken 15 min into perfusion. (NST = no special type; ILC = invasive lobular carcinoma; ER = oestrogen receptor; LVI = lymphovascular invasion; yp = post-chemotherapy pathological nodal stage.)

**Figure S3. Real-time electrophysiological and biochemical readings taken from the perfusate.**

(A, B) Cumulative ‘blood gas’ readings were measured from the perfusate surrounding the axillary lymph node and remained stable over time. The graphs show (A) the pCO_2_, pO_2_ and (B) the base excess, HCO_3_ and glucose readings over 12 h of perfusion (*n* = 13 patients). (C–K) Graphs show the change in (C) pH, (D) pCO_2_, (E) pO_2_, (F) base excess, (G) HCO_3_, (H) lactate, (I) glucose, (J) flow rate, and (K) pressure over 12 h of perfusion (*n* = 10 patients).

**Figure S4. Axillary lymph nodes (ALNs) perfused for 4 h.**

(A, B) A micrometastatic ALN and a cancer-replaced ALN that were both perfused for 4 h. (A, B) Top-left panels: ALN histology. Representative microscopy images of haematoxylin and eosin- and Ki67-stained ALN sections at x 100 and x 400 magnification (scale bars: 50 µm). Top-right panels: graphs show the flow rate (top) and perfusion pressure (bottom) over time. Bottom panels: ‘blood gas’ readings were measured from the ALN perfusate. Graphs show the change in pCO_2_, pO_2_, base excess, HCO_3_, lactate, glucose, and pH readings, calculated from the first reading taken 15 min into perfusion. (NST = no special type; ER = oestrogen receptor; LVI = lymphovascular invasion; yp = post-chemotherapy pathological nodal stage; p = pathological nodal stage.)

**Figure S5. Axillary lymph nodes (ALNs) perfused for 8 h.**

(A) Cancer-free ALN and (B) cancer-replaced ALN that were perfused for 8 h. (A, B) Top-left panels: ALN histology. Representative microscopy images of haematoxylin and eosin- and Ki67-stained ALN sections at x 100 and x 400 magnification (scale bars: 50 µm). Top-right panels: graphs show the flow rate (top) and perfusion pressure (bottom) over time. Bottom panels: ‘blood gas’ readings were measured from the ALN perfusate. Graphs show the change in pCO_2_, pO_2_, base excess, HCO_3_, lactate, glucose, and pH readings, calculated from the first reading taken 15 min into perfusion. (NST = no special type; ER = oestrogen receptor; LVI = lymphovascular invasion; p = pathological nodal stage.)


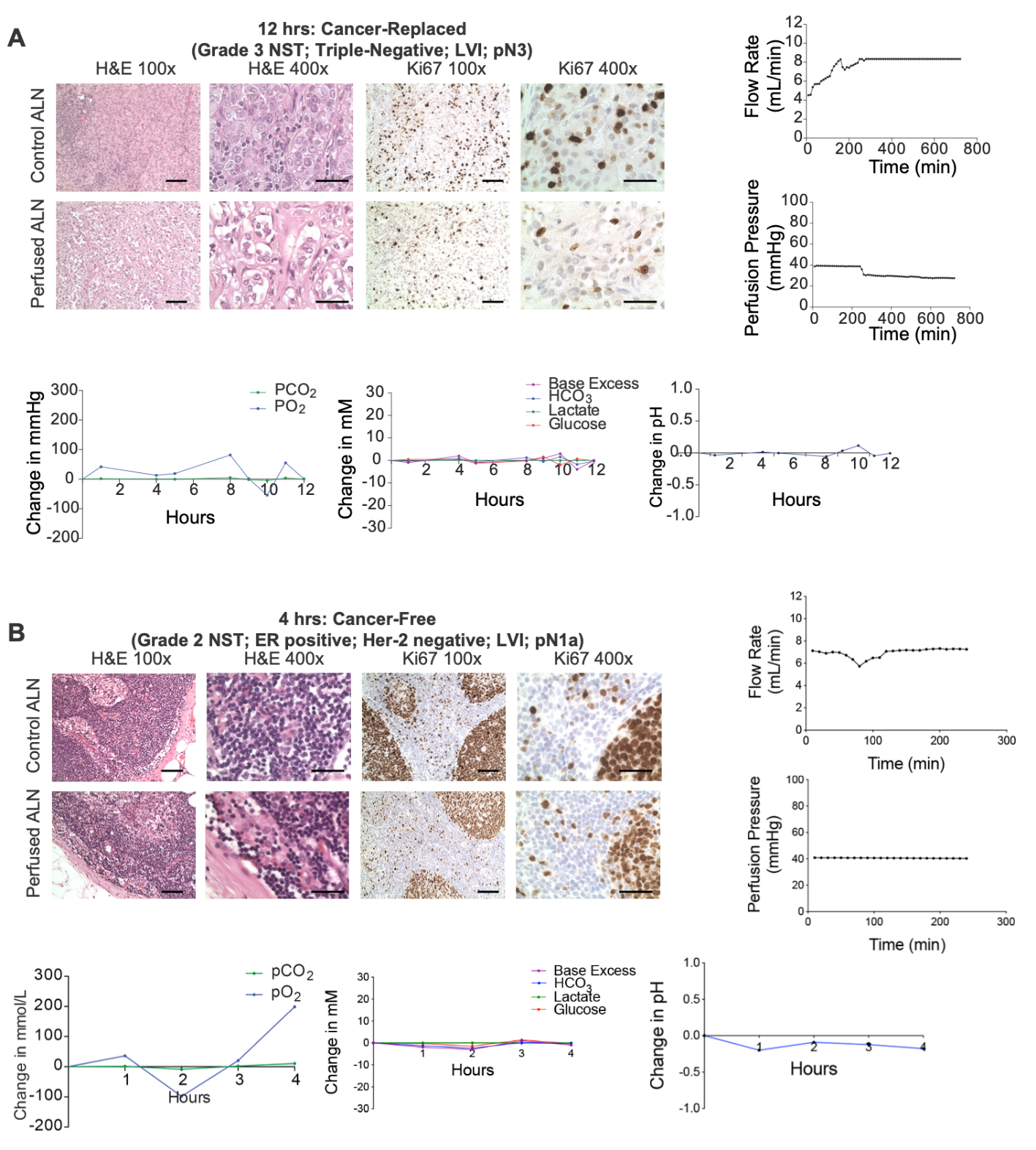


**Figure S6. Axillary lymph nodes (ALNs) perfused for 12 and 4 h.**

(A) Cancer-replaced ALN and (B) cancer-free ALN that were perfused for 12 and 4 h, respectively. (A, B) Top-left panels: ALN histology. Representative microscopy images of haematoxylin and eosin- and Ki67-stained ALN sections at x 100 and x 400 magnification (scale bars: 50 µm). Top-right panels: graphs show the flow rate (top) and perfusion pressure (bottom) over time. Bottom panels: ‘blood gas’ readings were measured from the ALN perfusate. Graphs show the change in pCO_2_, pO_2_, base excess, HCO_3_, lactate, glucose, and pH readings, calculated from the first reading taken 15 min into perfusion. (NST = no special type; ER = oestrogen receptor; LVI = lymphovascular invasion; p = pathological nodal stage.)

**Figure S7. Axillary lymph node (ALN) perfused for 24 h.**

A cancer-free ALN that was perfused for 24 h. Top-left panels: ALN histology. Representative microscopy images of haematoxylin and eosin- and Ki67-stained ALN sections at x 100 and x 400 magnification (scale bars: 50 µm). Top-right panels: graphs show the flow rate (top) and perfusion pressure (bottom) over time. Bottom panels: ‘blood gas’ readings were measured from the ALN perfusate. Graphs show the change in pCO_2_, pO_2_, base excess, HCO_3_, lactate, glucose, and pH readings, calculated from the first reading taken 15 min into perfusion. (NST = no special type; ER = oestrogen receptor; LVI = lymphovascular invasion; p = pathological nodal stage.)
